# Hydroxysafflor yellow A inhibits the proliferation, migration, and invasion of colorectal cancer cells through the PPARγ/PTEN/Akt signaling pathway

**DOI:** 10.1080/21655979.2021.2009965

**Published:** 2021-12-10

**Authors:** Dan Su, Chunye Lv

**Affiliations:** a Department of Gastrointestinal Surgery, The Sixth Affiliated Hospital of Sun Yat-sen University, Guangzhou, Guangdong, China; b Department of General Surgery, The Affiliated Jiangning Hospital with Nanjing Medical University, Nanjing, Jiangsu, China

**Keywords:** Colorectal cancer, hydroxysafflor yellow A, peroxisome proliferator-activated receptor γ

## Abstract

The natural compound Hydroxysafflor yellow A (HSYA) has been demonstrated to exert anti-cancer effect on multiple cancers. This study aimed to clarify the role of HSYA in inhibiting colorectal cancer (CRC) in vitro and the underlying mechanisms. Different concentrations of HSYA (0, 25, 50, and 100 μM) was exposed to HCT116 CRC cells, then cell proliferation, apoptosis, migration, and invasion were estimated by colony formation assay, TUNEL staining, wound-healing, and transwell assays, respectively. Western blotting assay was utilized to observe the expression of proteins involved in cell apoptosis, migration, and peroxisome proliferator-activated receptor γ (PPARγ)/PTEN/Akt signaling, including PCNA, Bax, Bcl-2, cleaved-caspase3, E-cadherin, N-cadherin, vimentin, PPARγ, and phosphorylated (p)-Akt. HCT116 cells that treated with 100 μM HSYA were also pre-treated with PPARγ antagonist, GW9662, or knockdown with PPARγ using short hairpin (sh)-RNA, to down-regulate PPARγ expression. Then, the above functional analysis was repeated. Results demonstrated that HSYA (25, 50 and 100 μM) significantly reduced HCT116 cell viability, but had no effect on the cell viability of human normal intestinal epithelial cell HIEC. HSYA also inhibited colony formation, migration, and invasion but promoted apoptosis of HCT116 cell in a concentration-dependent manner. Besides, the PPARγ/PTEN/Akt signaling was activated upon HSYA treatment. Finally, GW9662 and PPARγ knockdown blocked all the effects of HSYA on HCT116 cells. In conclusion, HSYA could exhibit anti-cancer effect on CRC via activating PPARγ/PTEN/Akt signaling, thereby inhibiting cells proliferation, migration, and invasion in vitro.

## Introduction

Colorectal cancer (CRC) is a common malignant tumor of the digestive tract, and it has become the second leading cause of cancer-related deaths all over the world. The latest data released by the World Health Organization indicate that the incidence and mortality of CRC in 2020 are approximately 24.8 and 12.0 per 100,000 [1]. Chemotherapy is one of the most effective adjuvant treatments for postoperative CRC [2]. However, the occurrence of multidrug resistance to chemotherapy is an important cause contributing to the failure of CRC treatment [3].

Hydroxysafflor yellow A (HSYA) is the major active ingredient in traditional Chinese medicine safflower extract, and it is also the water-soluble component with the highest content and the strongest pharmacological effect in safflor yellow [4]. Studies have suggested that HSYA exhibited multiple pharmacological effects such as anti-oxidation, anti-inflammatory, and anti-ischemia reperfusion injury [4–6]. In the last decade, emerging evidence implicated the anti-cancer effect of HSYA on various cancers, including liver cancer [7], esophageal cancer [8], lung cancer [9], hepatocellular carcinoma [10,11] and gastric carcinoma [12]. However, whether HSYA could inhibit CRC progression remains elusive.

Peroxisome proliferator-activated receptor γ (PPARγ) is a ligand-activated transcription factor of the nuclear receptor superfamily. PPARγ has been reported to play an important role controlling gene expression that related to a variety of physiological processes, thereby regulating cell proliferation, apoptosis and inflammation [13]. In addition, accumulative studies have concentrated on the indispensable role of PPARγ in various cancers, such as prostate, breast, hepatic, and bladder tumors [14,15]. The association between PPARγ polymorphisms and susceptibility CRC has also been suggested [16]. Intriguingly, a previous study showed that HSYA could trigger human gastric carcinoma cell apoptosis via activating PPARγ [12]. HSYA has also been illustrated to protect against hepatic fibrosis through PPARγ and its downstream signaling pathways activation [17,18]. These studies indicated that HSYA may play its role in alleviating diseases through regulating PPARγ pathway.

Therefore, we speculated that HSYA may exert anti-cancer effect on CRC via activating PPARγ/PTEN/Akt signaling. This study aimed to evaluate the effect of HSYA on CRC cells proliferation, migration, invasion, and apoptosis alongside the potential mechanisms.

## Materials and Methods

### Drugs

HSYA (cat. no. 78,281–02-4, HPLC ≥ 98%) was obtained from Shanghai Yuanye BioTech (Shanghai, China) and dissolved in sterile pyrogen-free water at a concentration of 100 mM as a working solution. The PPARγ antagonist, GW9662 (cat. no. SC9123) was purchased from Beyotime Biotechnology Research Institute (Shanghai, China). To inhibit PPARγ expression, cells were pre-treated with 2 μM GW9662 for 6 h [19].

### Cell culture

The human intestinal epithelial cell HIEC and CRC cell line HCT116 were purchased from the cell bank of the Chinese Academy of Sciences (Shanghai, China). All cells were cultured in DMEM medium (Gibco, USA) with 10% fetal bovine serum (FBS; Hyclone, USA), maintained at 37°C with 5% CO_2_ [20].

### Cell transfection

The PPARγ-targeting short hairpin RNA (shRNA) sequences were designed and synthesized by GenScript Biotech (Nanjing, China), then cloned into pSuper-retro-puro, named sh-PPARγ#1 and sh-PPARγ#2. Transfection of shRNAs was carried out using the Lipofectamine 2000 reagent (Invitrogen) according to the manufacturer’s instruction as previously reported [21]. At 48 h after infection, the knockdown efficiency was measured by RT-qPCR and Western blotting.

### Cell counting kit-8 (CCK-8)

The CCK-8 kit (Beyotime) was used to measure HIEC and HCT116 cell viability as previously reported [21]. Cells were seeded, at a density of 1 × 10^3^ cells per well, in 96-well plates, then incubated at 37°C and 5% CO_2_ overnight for confluence. Subsequently, cells were treated with 0, 25, 50 and 100 μM HSYA for 48 h, with or without 2 μM GW9662 pretreatment for 6 h. Then, the culture solution was discarded and 10 μL of CCK-8 solution was added. After incubation for 1 h, the cell viability was estimated using a microplate reader (Bio-Rad, USA) based on the absorbance at 450 nm.

### Colony formation assay

Colony formation was performed as previously reported [22]. HCT116 cells (1×10^3^ cells /well) were seeded in a 6-cm plate and cultured overnight for confluence, then cells were exposed to 0, 25, 50, and 100 μM HSYA for 48 h, with or without 2 μM GW9662 pretreatment for 6 h. Subsequently, the culture medium was replaced with fresh medium and cultured for another 14 days. Colonies were infiltrated with methanol for 10 min, stained with crystal violet for 10 min, counted and photographed (magnification x100).

### Terminal deoxynucleotidyl transferase (TdT) dUTP nick-end labeling (TUNEL) staining

Apoptosis of HCT116 cells was observed using a TUNEL apoptosis detection kit (Alexa Fluor 488) (Abcam, UK) according to the manufacturer’s instruction [23]. Briefly, after indicated treatment, cells were fixed by 4% paraformaldehyde for 0.5 h, incubated with hydrogen peroxide for 10 min, and then exposed to Triton X100 for 2 min, followed by being subjected to TUNEL staining reagents. The nuclei were counterstained with DAPI. Fluorescence microscopy (magnification x100; Olympus Corporation) was used to capture TUNEL-positive cells.

### Western blotting

Western blotting was performed as previously reported [23]. The HCT116 cells were lysed with RIPA lysis buffer according to the manufacturer’s protocol (Beyotime), and the protein concentration was determined using a BCA kit (Beyotime). Equal amount of proteins (40–50 μg) were separated by 10–12% SDS-PAGE and transferred onto PVDF membranes. After being blocked with 5% nonfat milk for 2 h, the blots were then incubated with primary antibodies at 4°C overnight. The membranes were then incubated with HRP secondary anti-mouse and anti-rabbit antibodies (Abcam; 1:10,000) for another 2 h at room temperature. Finally, the signals were detected using an ECL detection system (Bio-Rad Laboratories) and the protein levels were quantified using Image J software. Primary antibodies were purchased from Abcam Biotechnology (UK), including PCNA (1:1000), Bax (1:1000), Bcl-2 (1:1000), cleaved-caspase-3 (1:500), caspase-3 (1:1000), E-cadherin (1:500), N-cadherin (1:500), vimentin (1:500), PPARγ (1:1000), phosphorylated (p)-Akt (1:1000), Akt (1:1000), PTEN (1:1000) and GAPDH (1:5000). GADPH was used as the internal reference.

### Wound-healing assay and transwell invasion assay

To assess cell migration, HCT116 cells were seeded in 6-well plates at a density of 1 × 10^4^ cells/well and grown overnight for confluence. Then, the cells were scraped with pipette tips, washed with PBS and photographed. After that, fresh medium that supplemented with indicated drugs were added. After incubation for 48 h, three random areas of cells were photographed under a microscope (magnification ×200).

Transwell assay was employed to detect cell invasion capacity [24]. HCT116 cells were seeded into the upper chambers in FBS-free medium at a density of 1 × 10^5^ cells per chamber. The lower chamber was filled with 500 μL DMEM containing 10% FBS. After treatment, the invaded cells on the lower-chamber were fixed with formalin for 0.5 h and stained with crystal violet for 15 min, and finally photographed and counted by a light microscope (magnification x200).

### RNA extraction and quantitative RT-PCR (RT-qPCR)

Total RNA of treated HCT116 cells was extracted using TRIzol reagent. PrimeScript RT reagent Kit (Takara) was employed to perform reverse transcription according to the protocol. RT-qPCR was carried out using RT-PCR Kits (Takara) on a StepOnePlus Real-Time PCR system (Applied Biosystems, USA). Gene expression was analyzed via the 2^−ΔΔCT^ method [25] and primer sequences as follows: PPARγ forward: (5ʹ- CCAGAAGCCTGCATTTCTGC-3ʹ), reverse: (5ʹ-CACGGAGCTGATCCCAAAGT-3ʹ); GAPDH: forward: (5ʹ-AATGGGCAGCCGTTAGGAAA-3ʹ), reverse: (5ʹ-GCGCCCAATACGACCAAATC-3ʹ).

### Statistical analysis

All data represented three independent experiments and shown as mean ± standard deviation using GraphPad Prism 8.0. A one-way analysis of variance (ANOVA) with Tukey’s multiple comparison test was used to calculate the statistical differences. P < 0.05 was considered statistically significant.

## Results

### HSYA inhibits proliferation and induces apoptosis in CRC cells

**Figure 1.**
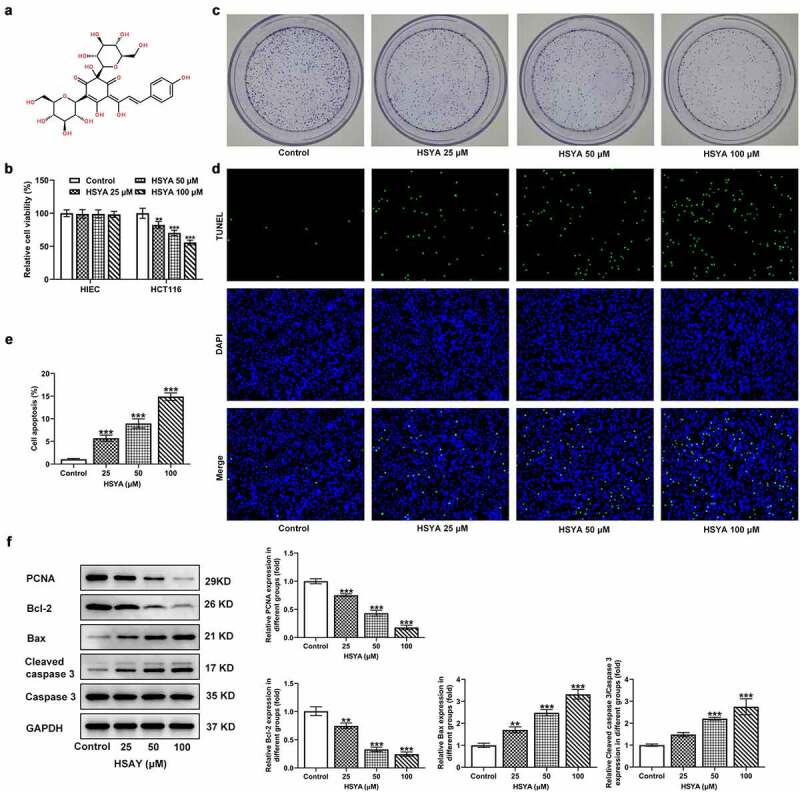
HSYA inhibits proliferation and induces apoptosis of HCT116 CRC cells

Firstly, we assessed the effect of HSYA on CRC cells viability. The chemical structure of HSYA was presented in Figure 1(a). The human normal intestinal epithelial cell HIEC and CRC cell line HCT116 were exposed to different concentrations of HSYA (0, 25, 50 and 100 μM) for 48 h, then cell viability was measured. As shown in Figure 1(b), HSYA did not affect HIEC viability but significantly impaired HCT116 viability in a concentration-dependent manner. This result confirmed the security of HSYA on normal intestinal epithelial cell as well as suggesting the potency of HSYA in inhibiting CRC. We next performed colony formation assay to observe the effect of HSYA on CRC cells proliferation. Results showed that different concentrations of HSYA (25, 50, and 100 μM) obviously reduced the colony number of CRC cells (Figure 1(c)). Then, the changes in cell apoptosis caused by HSYA were assessed by TUNEL staining and Western blotting assays. As illustrated in Figure 1(d–e), different concentrations of HSYA resulted in significant increase in apoptotic ratio of CRC cells (TUNEL-positive cells, green). Consistently, HSYA altered the expression of proteins involved in proliferation and apoptosis, manifesting with decreased PCNA and Bcl-2 expression, but increased Bax and cleaved caspase-3 expression caused by HSYA (figure 1(f)).

a, the chemical structure of HSYA (molecular formula C_27_H_32_O_16_, molecular weight 612 KD). b, HIEC and HCT116 cells were exposed to 0, 25, 50, and 100 μM HSYA for 48 h, then cell viability was measured using CCK-8 assay. c, representative images for colony formation assay of HCT116 cells that treated with 0, 25, 50, and 100 μM HSYA for 48 h (magnification, ×100). d and e, the apoptosis of HCT116 cells that treated with 0, 25, 50, and 100 μM HSYA, was observed by TUNEL staining (magnification, ×100). TUNEL-positive cells were stained with green and DAPI (blue) was used to stain the nucleus. f, the protein expression of PCNA, Bcl-2, Bax, cleaved-caspase 3/caspase 3 in HCT116 cells that treated with 0, 25, 50, and 100 μM HSYA, was detected by Western blotting assay. N = 3. **P < 0.01 and ***P < 0.001 vs Control. HSYA, Hydroxysafflor yellow A.

### HSYA suppresses migration and invasion of CRC cells

**Figure 2.**
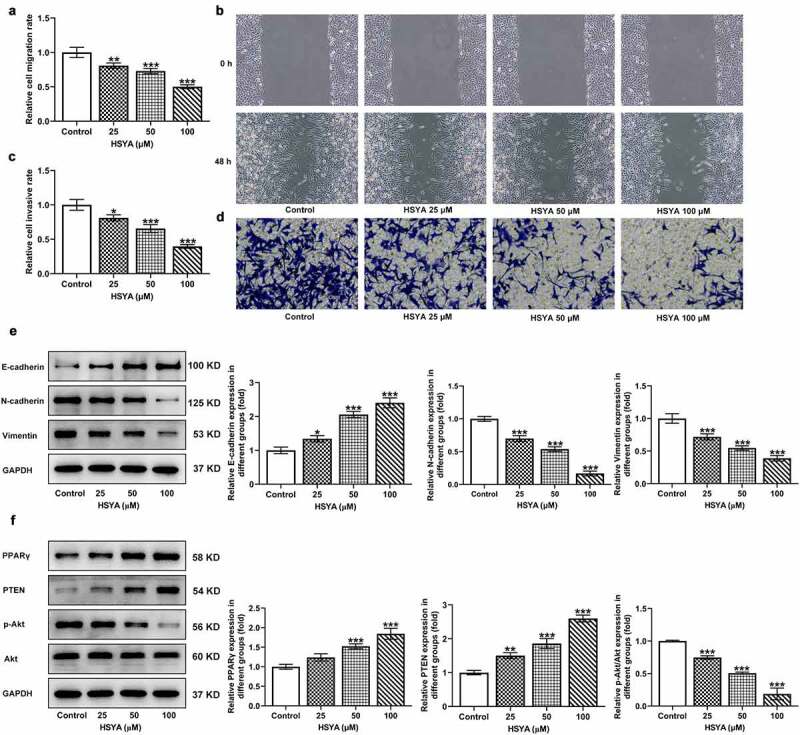
HSYA suppresses migration, invasion and activates PPARγ/PTEN/Akt signaling in HCT116 cells

Subsequently, the effects of HSYA on CRC cells migration and invasion were estimated. Results from wound-healing assay revealed that the cell migration rate was remarkably reduced upon HSYA (25, 50 and 100 μM) treatment (Figure 2(a–b)). Similarly, HSYA significantly decreased CRC cells invasive ability in transwell assay (Figure 2(c–d)). Moreover, different concentrations of HSYA led to significant increase in E-cadherin expression but decrease in N-cadherin and vimentin expressions (Figure 2(e)).

HCT116 cells were treated with 0, 25, 50, and 100 μM HSYA for 48 h, then (a and b) cell migration was measured by wound-healing assay (magnification, ×200); (c and d) cell invasion was detected by transwell assay (magnification, ×200); (e and f) the protein expression was assessed by Western blotting assay. N = 3. *P < 0.05, **P < 0.01 and ***P < 0.001 vs Control. HSYA, Hydroxysafflor yellow A.

### HSYA activates PPARγ/PTEN/Akt signaling in CRC cells

To uncover the possible mechanisms involving in HSYA, we investigated the effects of HSYA on PPARγ/PTEN/Akt signaling expression in CRC cells. We found that HCT116 cells that treatment with HSYA (25, 50 and 100 μM) showed higher expression of PPARγ and PTEN, but lower expression of p-Akt/Akt, when compared with control cells, suggesting the activation of PPARγ/PTEN/Akt signaling caused by HSYA (figure 2(f)). We therefore hypothesized that HSYA may exert its inhibitory effect on CRC via activating PPARγ/PTEN/Akt signaling.

### Inhibition of PPARγ blocks the effect of HSYA on CRC cells

**Figure 3.**
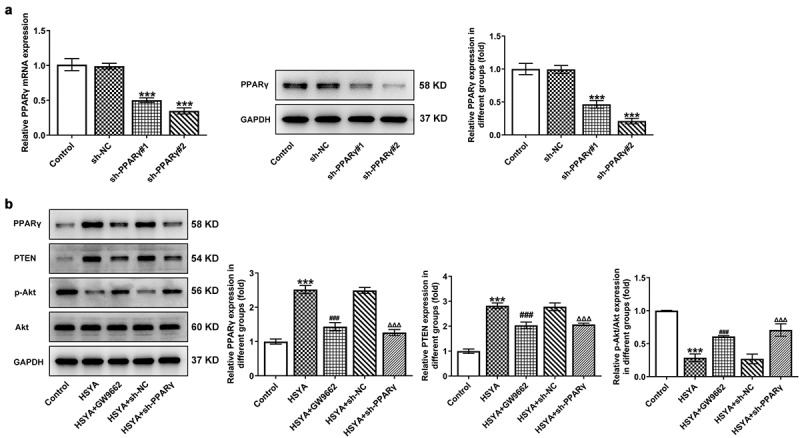
PPARγ down-regulation reverses the activation of HSYA on PPARγ/PTEN/Akt signaling

To further verify the above speculation, we knockdown PPARγ expression in HCT116 cells by shRNAs transfection, and sh-PPARγ#2 was selected for the following experiments based on the better transfection efficiency (Figure 3(a)). GW9662, a PPARγ antagonist, was also applied to inhibit PPARγ expression. As shown in Figure 3(b), GW9662 pre-treatment and sh-PPARγ transfection markedly reversed the expression of PPARγ, PTEN, and p-Akt/Akt, when compared with HSYA and HSYA + sh-NC group, respectively. These data indicated that the HSYA-mediated PPARγ/PTEN/Akt signaling activation was effectively inhibited by GW9662 and sh-PPARγ.

a, the mRNA and protein expression of PPARγ in control HCT116 cells or cells that transfection with indicated shRNAs. ***P < 0.001 vs sh-NC. b, the protein expression of PPARγ, PTEN and phosphorylated (p)-Akt/Akt in control HCT116 cells, cells that treated with 100 μM HSYA for 48 h with 2 μM GW9662 pre-treatment for 6 h or not, cells that transfection with indicated shRNAs then subjected to 100 μM HSYA treatment for 48 h. N = 3. ***P < 0.001 vs Control; ^###^P < 0.001 vs HSYA; ^ΔΔΔ^P < 0.001 vs HSYA + sh-NC. HSYA, Hydroxysafflor yellow A; sh, shRNA; GW9662, PPARγ antagonist.

**Figure 4.**
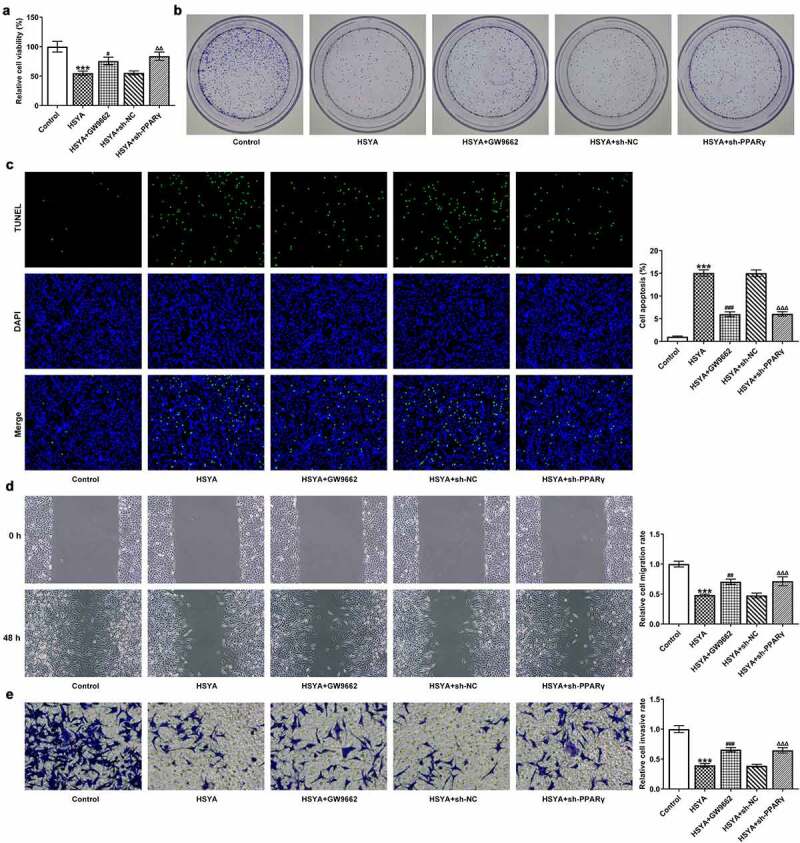
PPARγ down-regulation blocks the effect of HSYA on HCT116 CRC cells proliferation, apoptosis, migration and invasion

**Figure 5.**
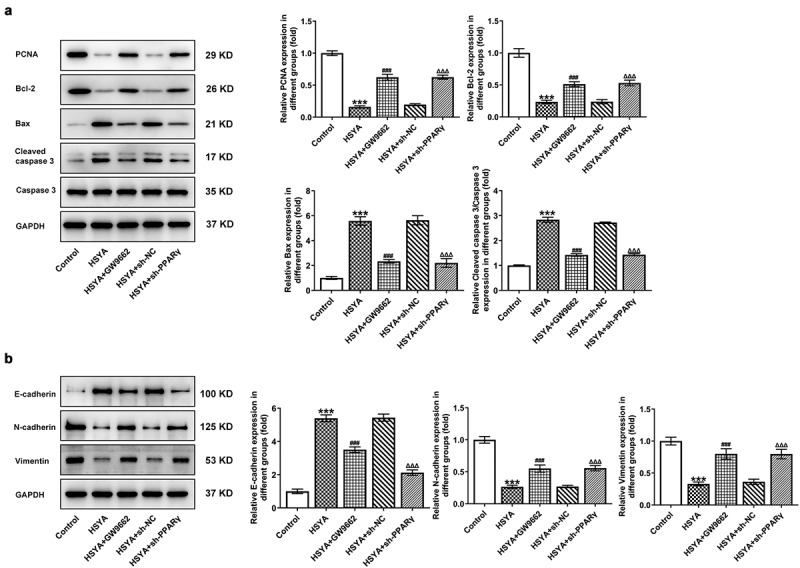
PPARγ down-regulation blocks the effect of HSYA on the expression of proteins related to proliferation, apoptosis and migration in HCT116 CRC cells

Finally, the alterations in HCT116 cells proliferation, apoptosis, migration and invasion, in the presence of GW9662 and sh-PPARγ were evaluated. Figure 4(a) shows that 100 μM HSYA treatment reduced cell viability, but additional GW9662 pre-treatment (HSYA + GW9662) enhanced cell viability compared with HSYA group. Meanwhile, HCT116 cells that transfection with sh-PPARγ had higher cell viability than cells that transfection with sh-NC, in the presence of HSYA treatment (Figure 4a). These results revealed that PPARγ inhibition markedly blunted the decrease in cell viability caused by HSYA. Consistent with these results, GW9662 pre-treatment and sh-PPARγ transfection significantly blocked the effects of HSYA on colony formation (Figure 4(b)), cell apoptosis (Figure 4(c)), migration (Figure 4(d)) and invasion (Figure 4(e)). Furthermore, the protein expression of PCNA, Bcl2, Bax, cleaved caspase3, E-cadherin, N-cadherin, and vimentin altered by HSYA, was also partially reversed by GW9662 pre-treatment and sh-PPARγ transfection (Figure 5). These data suggested that all the effects of HSYA on CRC cells could be reversed by PPARγ down-regulation.

HCT116 were treated with 100 μM HSYA for 48 h or not, pre-treated with 2 μM GW9662 for 6 h or transfected with indicated shRNAs, followed by 100 μM HSYA treatment for 48 h, then (a) cell viability was measured by CCK-8 assay; (b) colony formation was performed to observe cell proliferation (magnification, ×100); (c) TUNEL staining was utilized to explore cell apoptosis (magnification, ×100), TUNEL-positive cells were stained with green and DAPI (blue) was used to stain the nucleus; (d) cell migration was measured by wound-healing assay (magnification, ×200); (e) cell invasion was detected by transwell assay (magnification, x200). N = 3. ***P < 0.001 vs Control; ^#^P < 0.05, ^##^P < 0.01 and ^###^P < 0.001 vs HSYA; ^ΔΔ^P < 0.01 and ^ΔΔΔ^P < 0.001 vs HSYA + sh-NC. HSYA, Hydroxysafflor yellow A; sh, shRNA; GW9662, PPARγ antagonist.

HCT116 were treated with 100 μM HSYA for 48 h or not, pre-treated with 2 μM GW9662 for 6 h or transfected with indicated shRNAs, followed by 100 μM HSYA treatment for 48 h, then the protein expression was assessed by Western blotting assay. N = 3. ***P < 0.001 vs Control; ^###^P < 0.001 vs HSYA; ^ΔΔΔ^P < 0.001 vs HSYA + sh-NC. HSYA, Hydroxysafflor yellow A; sh, shRNA; GW9662, PPARγ antagonist.

## Discussion

In this study, we illustrated the promising anticancer function of HSYA in CRC in vitro, along with the molecular mechanisms underlying the concentration-dependent inhibitory effect of HSYA on cell proliferation, migration, and invasion and inducible effect on apoptosis in CRC cells. Importantly, we reported the activation of HSYA on PPARγ/PTEN/Akt signaling and PPARγ down-regulation reversed the anti-cancer effect of HSYA on CRC. Our results implied HSYA as a promising candidate for CRC therapy.

HSYA, a natural compound, produces anti-cancer effect via inhibiting tumor cells proliferation, migration, invasion, and angiogenesis, and inducing cells apoptosis [7–9,11,12]. In this study, we found that HSYA at 25, 50, and 100 μM, inhibited CRC cells proliferation and promoted apoptosis in a concentration-dependent manner. HSYA also decreased CRC cells migration and invasion rate in a concentration-dependent manner. The E-cadherin expression level was up-regulated, but N-cadherin and vimentin expressions were down-regulated upon HAYS treatment. E-cadherin, N-cadherin and vimentin are known to involve in epithelial–mesenchymal transition (EMT), which is a pivotal process during epithelial cancers acquire mesenchymal characteristics for invasion and metastasis [26,27]. The loss of E-cadherin expression together with increased expression of N-cadherin and vimentin were recognized as the marker of EMT, and were found in various cancers [28,29]. Collectively, our results reflected the actions of HSYA in inhibiting CRC aggravation in vitro.

We next intended to uncover the potential signaling pathways involving in the function of HSYA in CRC. Previous studies have indicated the activation of HSYA on PPARγ [12,17,18]. Here, we found that HSYA could increase PPARγ expression in CRC cells in a concentration-dependent manner. The PPARγ is a ligand-activated transcription factor of the nuclear receptor superfamily and is expressed in a variety of malignant tissues including prostate, breast, and colon. The role of PPARγ as a tumor suppressor in CRC has been documented [30]. Upregulation of PTEN was considered to be one of the molecular mechanisms for anticancer activity of PPARγ [31]. It has been demonstrated that PPARγ activation upon ligands binding, upregulated PTEN expression in CRC cells [32]. Another study also showed that the PTEN gene expression in CRC cells was enhanced after treatment with rosiglitazone, a synthetic ligand for PPARγ [33]. PTEN mediates cell proliferation, migration, and survival through inhibiting phosphatidylinositol 3 kinase (PI3K)/Akt signaling cascades. PPARγ agonist efatutazone and gefitinib have been indicated to synergistically inhibit the proliferation of EGFR-TKI-resistant lung adenocarcinoma cells via the PPARγ/PTEN/Akt pathway [34]. PPARγ/PTEN/Akt was also suggested to induce apoptosis in differentiated human embryonic stem cells [35]. We therefore shed light on the PPARγ/PTEN/Akt signaling in HCT116 CRC cells after HSYA treatment. In accordance with our speculation, results showed that PPARγ and PTEN expression was markedly increased, and p-Akt expression was decreased by HSYA treatment.

To further validate the involvement of PPARγ/PTEN/Akt signaling in the effect of HSYA on CRC. In addition to 100 μM HSYA treatment, HCT116 cells were also exposed to PPARγ antagonist GW9662, or knockdown with PPARγ by shRNA transfection, to down-regulate PPARγ expression in HSYA-treated HCT116 cells. Then the changes in cells development processes were investigated. We found that GW9662 pre-treatment or PPARγ shRNA transfection effectively down-regulated PPARγ and PTEN expression, but up-regulated p-Akt expression, indicating the activation of PPARγ/PTEN/ Akt signaling induced by HSYA, was inhibited by PPARγ down-regulation. Importantly, all the effects of HSYA on cell proliferation, apoptosis, migration and invasion were reversed by both GW9662 treatment and PPARγ knockdown. Akt signaling is an important event in colorectal carcinogenesis, activation of it upon phosphorylation can contribute to cell proliferation and tumor progression by modulating cellular events, such as cell growth, adhesion, migration and survival [36,37]. As a result, it was speculated that the inhibition of Akt activation that regulated by PPARγ up-regulation was responsible for the cancer suppressor role of HSYA in CRC. However, there are some limitations in this study. Firstly, this study was performed on only one CRC cell line, more CRC cell lines as well as animal models will be utilized to further confirm our findings. Besides, it can be observed that both PPARγ antagonist treatment and PPARγ knockdown did not cancel the anti-cancer effect of HSYA totally, suggesting the involvement of other pathways in the HSYA-mediated CRC inhibition, which need to be clarified in the future research.

## Conclusion

To conclude, HYSA suppressed proliferation, migration, invasion, and EMT in HCT116 CRC cells by activating the PPARγ/PTEN/Akt signaling pathways, implying that HYSA may be a promising candidate for CRC therapy.

## Highlights

- Hydroxysafflor yellow A inhibits colorectal cancer cells proliferation.
- Hydroxysafflor yellow A suppresses colorectal cancer cells migration and invasion.
- Hydroxysafflor yellow A inhibits colorectal cancer via PPARγ/PTEN/Akt signaling.
